# Health status of recently arrived asylum seekers in their host country: results of a cross-sectional observational study

**DOI:** 10.1186/s12889-022-14095-8

**Published:** 2022-09-06

**Authors:** Jérémy Khouani, Léo Blatrix, Aurélie Tinland, Maeva Jego, Gaëtan Gentile, Guillaume Fond, Anderson Loundou, Marilou Fromentin, Pascal Auquier

**Affiliations:** 1 Aix-Marseille Univ, – CEReSS UR3279-Health Service Research and Quality of Life Center, Marseille, France; 2 Department of General Practice, Aix-Marseille Univ, Marseille, France; 3 Department of Psychiatry, Assistance Publique - Hôpitaux de Marseille, Marseille, France; 4 Department of Public Health, Assistance Publique - Hôpitaux de Marseille, Marseille, France

**Keywords:** Asylum seeking, Health status, Prevalence, Mental health

## Abstract

**Background:**

The World Health Organization (WHO) considers that the heterogeneity of concepts and definitions of migrants is an obstacle to obtaining evidence to inform public health policies. There is no recent data on the health status of only asylum seekers who have recently arrived in their Western host country. The purpose of this study was to determine the health status of asylum seekers and search for explanatory factors for this health status.

**Methods:**

This cross-sectional observational study screened the mental and somatic health of adult asylum seekers who had arrived in France within the past 21 days and went to the Marseille single center between March 1 and August 31, 2021. In order to study the explanatory factors of the asylum seekers' health status, a multivariate analysis was performed using a logistic regression model to predict the health status. Factors taken into account were those significantly associated with outcome (level < 0.05) in univariate analysis.

**Results:**

In total, 419 asylum seekers were included and 96% CI95%[93;97.3] had at least one health disorder. Concerning mental health, 89% CI95% [85.1;91.4] had a mental disorder and in terms of somatic health exclusively, 66% CI95% [61.4;70.6] had at least one somatic disorder. Women were more likely to have a somatic disease OR = 1.80 [1.07; 3.05]. We found a statistically significant association between the presence of at least one disorder and sleeping in a public space OR = 3.4 [1.02;11.28] *p* = 0.046. This association is also found for mental disorders OR = 2.36 [1.16;4.84], *p* = 0.018.

**Conclusions:**

Due to the high prevalence of health disorders our study found**,** asylum seekers are a population with many care needs when they arrive in their host country. The main factors linked to a poor health status seem to be related to a person’s sex, geographical origin and sleeping in a public space.

## Introduction

Migration flows have been permanent since the 19th century [1]. In 2020, the United Nations estimated the number of international migrants worldwide at 281 million [2]. In Europe, almost 10% of the population is migrants from outside Europe [3]. Violence and political conflicts are among the reasons that have led to these population displacements. Many of them had to leave their country of origin [3]. This fact raises the question of appropriate social and health care [2]. However, the World Health Organization (WHO) considers that the heterogeneity of concepts and the definitions of migrants is an obstacle to obtaining evidence to inform public health policies. Thus, it recommends distinguishing refugees from asylum seekers [3]. Asylum seekers are migrants who recently arrived in their host country and whose administrative situation is being examined. They do not have the same access to healthcare or the same rights as refugees. Indeed, refugees benefit from international protection and their status has been recognized by the host country. People who have recently arrived and are seeking asylum have an unstable administrative (residence permit) and social (housing, work) situation that may affect their health status [4]. This is essential when we take into consideration that the WHO considers that legal status is one of the most important determinants of migrants' access to health services in a country [3]. The reception of people seeking asylum and the management of their health needs is an international issue and a source of recurrent crisis. From 2010 to 2020, the number of asylum seekers has increased by five-fold [5]. In 2020, the United Nations High Commissioner for Refugees (UNHCR) estimated that 4.1 million people were seeking asylum in the world [5] and 535,000 in Europe [6]. In France in 2021, 78,372 adult-age people filled out an initial asylum application, a 26.4% increase compared to 2020 [7].

Regarding the mental health of exiles, a literature review informs us that 31.5% of them suffer from post-traumatic stress disorder (PTSD), 31.5% from depression and 11.1% from anxiety disorders [8]. However, these data are taken from studies that do not make a distinction between refugees and asylum seekers. We found 4 studies about asylum seekers exclusively (1 in Italy, 1 in Sweden, 2 in Germany). The prevalence of PTSD ranged from 9.5% [9] to 60.7% [10], that of depression from 21.3% [11] to 67.9% [10] and that of anxiety from 41% [12] to 59.3% [10]. The studies in Italy and Sweden were only able to include asylum seekers from a limited geographical area. These results are too disparate to help public health policies.

Concerning the overall somatic health of asylum seekers only, a German retrospective study conducted in 2015 found a prevalence of HIV and hepatitis B infections at 0.3%, 3.3% [13] respectively. Two others found a prevalence of less than 1% of tuberculosis (TB) disease in this population [14, 15].

Regarding non-communicable diseases, a literature review published in 2014 [16] found only 8 studies. These studies do not distinguish refugees from asylum seekers either. Moreover, they were conducted before 2010 and only among the populations originating from Iraq, Palestine or North Korea, which are not representative of asylum seekers living in Western countries. Since 2014, non-communicable disease data was about refugees and was conducted in non-Western countries (Jordan, Lebanon) on populations with restricted geographic origins (Syria, Iraq, Afghanistan).

These missing data about their health status and its explanatory factors are a hindrance to the development of efficient strategies for the management of these populations within the health systems of Western countries [3].

The primary objective of this study was to describe the health status of asylum seekers who have recently arrived in France. The secondary objective was to investigate potential explanatory factors for the health status of asylum seekers.

## Materials and methods

This study was approved by one of the French Research Ethics committees (N°IRB: IORG0009855) under the authority of the Ministry of Research on November 27, 2020 (Approval number 20.10.09.57715). All methods were performed in accordance with the Declaration of Helsinki regarding ethical aspects, information and consent of subjects to participate and data publication. Written informed consent was obtained from all subjects and/or their legal guardian(s). Its registration number on ClinicalTrials.gov is NCT05423782 (Registration 21/06/2022).

### Design

We conducted a single-center, cross-sectional, observational epidemiological study.

### Population

People wishing to apply for asylum in France must register at the reception platform for asylum seekers (RPAS) in their region of arrival. Our study was conducted in Marseille during this initial registration process for asylum seekers. Participants were screened. The investigators of our study were positioned inside the reception platform (RPAS) in a confidential space, on the days when the RPAS was open, i.e. 4 and a half days a week between March 1, 2021 and August 31, 2021. We consecutively included all individuals who met our inclusion criteria and consented to participate in this study. Inclusion criteria took into account people who were over the age of 18, registering with the Marseille RPAS and had an initial asylum application receipt (within the past 21 days or an appointment at the prefecture to obtain this initial receipt). Individuals with cognitive (dementia) or mental (mental retardation) impairments limiting comprehension or oral expression (dysphasia, aphasia) were excluded. Professional telephone interpretation was used for each non-native speaker to ensure that the lack of French language skills did not limit the understanding and informed consent of the study. With the help of these interpreting services, all subjects consented to participate in the study by signing a form following the delivery of clear, fair and appropriate information in their language of use.

### Data collection

A face-to-face questionnaire was filled in using Redcap software (www.project-redcap.org). This questionnaire included data about age, sex, education level, marital status (single, children, spouse), country of origin, type of asylum procedure (Regular, accelerated or Dublin [17]) and housing status. 5 categories of housing status were initially collected (public space, squat, acquaintance's house, self-catering hotel with charge, state accommodation scheme). We chose these 5 categories to reflect French situation about the most common types of housing of asylum seekers. Their correspondence to the European ETHOS Typology on Homelessness and Housing Exclusion [18] is as follows: Public space (ETHOS 1.1), Squat (ETHOS 8.2 or 8.3 or 11), acquaintance’ house (ETHOS 8.1), State accommodation scheme (ETHOS 2 or 3 or 4 or 5 or 7).

#### Somatic health

To assess participants' somatic health, we administered the Cumulative Illness Rating Scale (CIRS) used to assess comorbidity, collected data regarding tobacco use (Pack years-number), performed a standardized biological assessment and proposed a chest X-ray for TB screening at the anti-tuberculosis center (CLAT).

The CIRS is a comprehensive measure of multimorbidity previously validated on homeless people [19–21]. Each item is assigned a severity score by means of assessing the impact on the patient: 1 (no problem), 2 (current mild problem or past significant problem), 3 (moderate disability or morbidity), 4 (severe problem), 5 (extremely severe or life-threatening problem). The CIRS classifies all items into 14 body systems (including one for mental disorders) to obtain a final cumulative score. Information regarding treatments taken according to the reported problems was also collected. To describe the disorders by system, we merged the following CIRS diseases categories together: cardiac and hypertension, hepatic and gastrointestinal, renal and urological.

Our standardized biological work-up included screening for the main infectious diseases: HIV (confirmed by 2 samples including a western blot), hepatitis B and C (viral loads), syphilis (TPIE and VDRL serology), strongyloidiasis or bilharzia parasitosis (serologies), gonococcal or chlamydial urethritis (detected by urinary PCR). Concerning vaccinations, we looked for protection against tetanus (immunization if tetanus antibody > 1 IU/ml) and hepatitis B (Serology with HBsAb, HBsAg, HBcAb). In addition, the biological work-up screened for the following non-infectious diseases: diabetes (fasting blood glucose level > 2 g/l), renal dysfunction (creatinine > 104umol/l) and dysthyroidism (hyperthyroidism if thyroid stimulating hormone (TSH) < 0.270 mIU/L, hypothyroidism if TSH > 4.20 mIU/L). Finally, we looked for pregnancy in women of childbearing age (Blood HCG > 5 IU/l).

People were considered to have a metabolic disease if they were identified with the CIRS or if they had diabetes or dysthyroidism identified by a blood test. People with a urinary tract disease were those for whom this type of disease was identified with the CIRS or those for whom renal dysfunction was detected by a blood test.

People were considered to have a morbidity if they had at least one CIRS item scored at 2 or higher and/or if they had a disease detected with a blood test and/or x-ray.

#### Mental health

Mental health status was assessed by the Refugee Health Screener (RHS-15) and by the CIRS item that detects a mental disorder.

The RHS-15 is a valid [22, 23] 15-item instrument to detect Post-traumatic stress disorder (PTSD), anxiety or depression symptoms in asylum seekers and refugees. 14 items are rated on a 5-point Likert scale (0 = not at all to 4 = extremely). The last item assesses the general ability to handle stress on a 5-point Likert scale and a distress thermometer (DT) ranging from 0 to 10. A screening result is positive if the sum of the first 14 items ≥ 12 or if the DT is ≥ 5. The RHS-15 is not disorder specific and does not provide a diagnosis. In case of a positive result, we can conclude that there is a symptomatology that could correlate to anxiety, depressive or post-traumatic stress disorders.

In order to identify patients with a psychiatric disorder not included in those detected by the RHS-15, we also identified those for whom the CIRS Psychiatric Disorder item reached a 2 or higher mark.

People were considered to have a mental disorder if they had a positive RHS-15 screening and/or for whom the CIRS Psychiatric Disorder item reached a 2 or higher mark.

Patients were considered sick if they had at least one somatic or mental disorder.

### Data analysis

Statistical analysis was performed using IBM SPSS Statistics version 20 (IBM SPSS Inc., Chicago, IL, USA). Results were expressed as proportions with 95% confidence intervals (CI) or ± SD means (standard deviation) in order to estimate from the results of our sample the corresponding figures for all asylum seekers living in France. The association of outcomes with categorical variables was evaluated using the Chi square or Fisher’s exact test, and the student t-test or Mann–Whitney U test for continuous ones, as appropriate. A multivariate analysis was performed using a logistic regression model to predict health status. Factors taken into account were those significantly associated with outcome (level < 0.05) in univariate analysis. Calibration was assessed using the Hosmer–Lemeshow goodness-of-fit test to evaluate the discrepancy between observed and expected values. A two-sided *P* value of less than 0.05 was considered statistically significant.

## Results

### Participants

Between March 1, 2021, and August 31, 2021, 1,953 individuals came to register at the RPAS. The participation time was 1 h and 30 min; we were able to include 4 people per day, so that we had a sample of 419 asylum seekers, counting the days our interviewers were absent.

The sociodemographic and health status characteristics of the asylum seekers are reported in Table 1.

**Table 1:** Sociodemographic and health status characteristics

Description		Population	Pct. (%)	CI95%
A. Sociodemographic characteristics				
Age		*n* = 419		
Mean/SD/Range		30.3/9.7/18–80		
Sex^a^				
Men		285	68%	[63.6;72.5]
Women		133	32%	[27.5;36;4]
Geographical origin		*n* = 398^b^		
West Africa		159	40%	[35.3;44.8]
Magreb		62	16%	[12.3;19.5]
Central Asia		61	15%	[12.1; 19.2]
Europe		49	12%	[9.4;15.9]
Middle east		46	12%	[8.8;15.1]
Rest of Africa		21	5%	[3.4;7.9]
Asylum procedure		*n* = 363^b^		
Dublin		201	55%	[50.1;60.6]
Accelerated procedure		48	13%	[9.9;17.2]
Regular		111	31%	[25.9;35.6]
Other		3	0,8%	[0.2;2.4]
Accompaniement		*n* = 411^b^		
Alone		240	58%	[53.5;63.2]
Children		41	10%	[7.3;13.3]
Spouse		55	13%	[10.2;17.1]
Other		75	18%	[14.6;22.3]
Housing		*n* = 417^b^		
Public space		188	45%	[40.2;50.0]
Acquaintance's house		162	39%	[34.3;43.6]
State accommodation scheme		49	12%	[9;15.2]
Squat		10	2%	[1.3;4.4]
Self-catering hotel with charge		5	1%	[0.4;2.9]
Other		3	1%	[0.1;2.2]
Education level		*n* = 408^b^		
Less than primary		67	16%	[13,1;20.3]
Less than secondary		248	61%	[55.9;65.4]
High school		52	13%	[9.8;16.4]
More than highschool		41	10%	[7.5;13.4]
B. Health status characteristics of the study sample				
Health disorder		400	96%	[93;97.3]
1 health disorder		145	35%	[31.5;41.2]
2 health disorder		135	32%	[29.1;38.6]
3 health disorder		65	16%	[12.9;20.2]
4 health disorder or more		55	13%	[10.5;17.5]
Psychiatric disorder		371	89%	[85.1;91.4]
*of which specifically*				
Anxiety, depression, PTSD (RHS15)		368	88%	[84.3;90.8]
Somatic disorder (at least one)		277	66%	[61.4;70.6]
Infectious disease (at least one)		95	23%	[18.8;26.9]
*of which specifically*				
HIV		2	0,5%	[0;1.9]
Positive HBV viral load		20	5%	[3.2;7.6]
Positive HVC viral load		4	1%	[0.3;2.6]
Anguillulosis		39	10%	[7.2;13.1]
Bilharziasis		40	10%	[7.4;13.4]
Cardiac		35	8%	[5.9;11.4]
*of which specifically*				
Hypertension		13	3%	[1.7;5.3]
Hematologic		45	11%	[7.9;14.1]
Respiratory		158	38%	[33.1;42.5]
*of which specifically*				
Tobacco consumption		147	35%	[30.5;39.8]
Ophtalmological and otholaryngologic		37	9%	[6.3;11.9]
Hepatic and gastrointestinal		73	17%	[13.9;21.4]
Renal and urological		18	4%	[2.6;6.7]
Musculoskeletal		49	12%	[8.8;15.2]
Neurological		43	10%	[7.7;13.6]
Endocrine and metabolic		37	9%	[6.3;11.9]
*of which specifically*				
Diabetes		5	1%	[0.4;2.9]
Hypothyroidism		10	3%	[1.2;4.4]
Hyperthyroidism		3	1%	[0.1;2.2]
Pregnancy	*n* = *119*	27	23%	[16;31.1]

^a^ Whose sex is undertermined *n* = 1

^b^ Number of participants changed due to missing data about sociodemographic characteristics for some individuals

The average age was 30.29 (± 9.7) years, 68% were male. Regarding geographical origin, the most represented region was West Africa (29.4%). Regarding housing, 44.9% had no accommodation and slept in the street or a public space.

More than 95% of participants had at least one health disorder.

Concerning mental health, almost 9 out of 10 had a psychiatric disorder detected by RHS-15 and only 3 individuals had another mental disorder than those detected by RHS-15.

On the other hand, more than 66% participants had at least one somatic disorder and 22.7% at least one infectious disease.

The HIV prevalence was 0.5% (2), all from West Africa. The prevalence of a positive HBV viral load was 4.9% (20). Of these 20, 14 were in the Dublin procedure as were the two people who had HIV and one who had HCV. Only 29 persons (6.9%) were vaccinated against hepatitis B while 226 asylum seekers (55.3%) had an indication for a hepatitis B vaccination due to accumulating HBsAg, HBsAb and HBcAb. Only 39 included subjects were screened for N. gonorrhoeae and C. trachomatis infections due to a lack of access to toilets in our study area and therefore to urine sampling. Only one of these had a C. trachomatis infection and none had a N. gonorrhoeae infection. Concerning tetanus, 31.6% of the asylum seekers had an indication for a tetanus vaccination booster due to the seronegativity of the anti-tetanus antibodies. Regarding the tuberculosis screening, 236 out of 386 persons (61.1%) refused it. Of the 150 people who were referred to the CLAT only 13 actually went. Of these 13, none had tuberculosis.

In the matter of non-communicable diseases, 8.4% (24) of them had a cardiovascular problem and 3.2% (13) were taking antihypertensive treatment before arriving in France. 147 asylum seekers (34.8%) were active smokers.

The prevalence of pregnancy was 22.7% (27 positive HCG samples / 119 samples taken from female asylum seekers of childbearing age). Among them, 25.9% were sleeping in a public space (7) and 63% were in the Dublin procedure (17). 92.6% (25)  had a mental disorder and 1 person had active hepatitis B and was HIV positive.

Table 2 (Univariate analysis) describes the differences found in the univariate analysis for each sociodemographic data according to whether the person was sick or not, had a mental disorder or not, had a somatic disease (infectious or non-communicable disease) or not.

**Table 2:** Univariate analysis

		**Sick**			**Mental disorder**			**Somatic disease**			**Infectious disease**			**Non-infectious disease**		
		Yes	No	Test	Yes	No	Test	Yes	No	Test	Yes	No	Test	Yes	No	Test
Age (mean)		30.32	29.58	0.745	30.28	30.38	0.948	30.64	29.61	0.308	28.03	30.95	0.003	31.2	29.2	0.045*
Sex	Men	270	15	0.158	248	37	0.1	178	107	0.016*	61	224	0.344	148	137	0.002*
	Women	130	3		123	10		99	34		34	99		90	43	
Geographical Origin	Europe	47	2	0.969	44	5	0.022*	34	15	0.089	2	47	< 0.000*	34	15	0.097
	Middle east	44	2		39	7		28	18		3	43		27	19	
	West Africa/Nigeria	152	7		147	12		117	42		67	92		92	67	
	Rest of Africa	20	1		18	3		12	9		6	15		8	13	
	Central Asia	57	4		46	15		33	28		7	54		29	32	
	Maghreb	60	2		57	5		40	22		5	57		39	23	
Asylum procedure	Regular	107	4	0.231	95	21	0.581	73	38	0.391	20	138	0.002*	64	47	0.947
	Dublin	191	10		180	16		138	63		63	91		112	89	
	Accelerated	43	5		42	6		28	20		5	43		27	21	
Marital status	Single	229	11	0.65	215	25	0.72	161	79	0.362	62	178	0.086	136	104	0.735
	Children	38	2		35	5		26	14		6	34		24	16	
	spouse	54	1		48	7		39	16		16	39		32	24	
	other	70	5		64	11		43	32		11	64		38	37	
Accommodation	Public space	183	5	0.131	174	14	0.025*	132	56	0.138	50	138	0.092	110	78	0.591
	other	216	13		196	33		145	84		45	184		128	101	
Study Level	never been to school	65	2	0.638	60	7	0.825	46	21	0.15	27	40	< 0.000*	34	33	0.092
	Lower than High school	237	11		217	31		164	84		56	40		141	107	
	High school	48	4		46	6		28	24		5	192		26	26	
	More than high school	39	2		38	3		31	10		5	36		30	11	

^*^ Statistically significant

For overall health, there is no statistically significant difference between the health status of men and women. However, with regard to somatic diseases, there is significant higher morbidity among women, which is also the case for non-infectious diseases (*p* = 0.02).

For mental health conditions, we reported significant differences in terms of housing and geographical origin.

People who slept in public spaces after arriving in their host country more often had a mental disorder with a statistically significant difference (*p* = 0.025). Also, people from West Africa more often had a mental (*p* = 0.022) disorder and infectious disease (*p* < 0.000).

Multivariate analyses adjusted for age, gender, geographical origin, and housing status were conducted.

The trend towards poorer health status for women was confirmed after these adjustments. They were more likely to have a somatic disease OR = 1.80 [1.07; 3.05] or a non-infectious disease OR = 2.28 [1.36;3.80].

We also found a statistically significant association in the multivariate analysis between the presence of at least one disorder and sleeping in a public space OR = 3.4 [1.02;11.28] *p* = 0.046. This association is also found for mental disorders OR = 2.36 [1.16;4.84], *p* = 0.018.

West African origin was significantly associated with the presence of an infectious disease OR = 5.64 |1.92; 16.54] *p* = 0.002).

## Discussion

All people requesting asylum in France and residing in the departments of Bouches-du-Rhône, Vaucluse, Hautes Alpes and Alpes de Hautes Provence must register at the Marseille asylum seekers reception platform. During our study period, we included 21.45% of the 1,953 asylum seekers registering for an initial asylum application on this platform.

Our sample is representative of asylum seekers living in France in terms of age, gender, geographical origin, and family status [7, 24].

One of the originalities of our study was to include asylum seekers in the only place where the selection bias could be limited: the reception platform they had to go through in regards to the procedure. The study is not restricted to asylum seekers receiving accommodations or consulting at a care center. Also, the inclusions took place less than 21 days after their arrival in the host country in order to have a homogeneous population that would provide an overview of the health status of people when they first arrive in their host country, limiting the collection of pathologies likely to be developed after their arrival, in particular due to their precarious social living conditions [25].

The health status reported seems poorer than that of the population of their Western host country. Indeed, 71.1% of men and 66.3% of women in the European Union perceive their health as good or very good [26]. Regarding chronic infectious diseases, the prevalence of hepatitis B in our sample is almost 10 times higher than that existing among the general French population [27] and for HIV and HCV, almost 2 times higher [28, 29]. It is also slightly higher than the prevalence described for refugees (2.04%, 0.4% et 0.41% respectively for HIV, hepatitis B and C [30]). The respective prevalence of depression and anxiety disorders in the world is 4.4% and 3.6% [31] and 11% of adults in the European Union had symptoms of psychological distress [32]. Our results show that the mental health status of asylum seekers is worse than the general population, which may be partly due to exposure to a traumatic event. We also found in the literature a 59% prevalence among asylum seekers of at least one disorder among PTSD, depression or anxiety [11]. This figure is lower than our study and can be explained by the fact that this figure has been established with a true diagnosis, whereas our study focused on screening and only noted the existence of a symptomatology. Moreover, as the respective prevalence rates in the literature for PTSD, depression and anxiety in asylum seekers are around 30% [8] it is not surprising to find a prevalence of nearly 90% for the symptomatology of one of these 3 disorders.

In terms of care needs, almost all our sample (95.5%) had at least one health problem and therefore a need to access the healthcare system.

Taking into account the deteriorated state of health for the asylum seekers reported, we can suspect that policies restricting access to healthcare for this population generate additional costs for healthcare systems [32, 33].

The social and administrative status may have complicated the care pathway for people in need of care. For example, 1 in 4 pregnant women slept in public spaces. Also, 17 individuals were in the Dublin procedure and had one of the following conditions: HIV, hepatitis B or C. Their asylum application was not examined in France, but in the first European country where they applied for asylum or whose borders they crossed. Unless their type of procedure is reclassified, these people are supposed to be removed from France and taken to the European country in which the examination of their asylum application will take place.

The care pathways for these individuals are weakened by the risk of a disruption due to the remoteness of the territory. These disruptions in care could lead to serious consequences for their health as well as for others with the risk of transmission.

In our study, housing and administrative status have an impact on asylum seekers' health status. This link between post-migratory difficulties and mental disorder has already been described among Nigerian asylum seekers [34]. Thus, reducing the number of asylum seekers sleeping in public spaces could lead to a decrease in their mental disorders. These findings should be seen in the context of the high number of asylum seekers living in a public space in our study even though French law provides for access to accommodation for each of them [35–37]. This is due to a discrepancy between the number of places available in the French state accommodation scheme play and the total number of asylum seekers. Despite an annual increase of accommodation places in reception centers for asylum seekers [38], the parallel increase in the total number of new asylum seekers is biggest.

Western countries' health systems should be prepared to screen all their asylum seekers for mental health problems as well as provide care for a significant number of them. Particular attention should be paid to vaccinations against hepatitis B, considering the high prevalence of active hepatitis B among this group and the low number of them vaccinated against this disease. Regarding care programs for asylum seekers, they should not be limited to mental health or infectious diseases.

A small number of people were screened for tuberculosis despite the geographical proximity of the CLAT to our study site. This allows us to formulate the hypothesis that a healthcare offer in a unique site, delocalized from the usual services, located as close as possible to the places where the targeted populations live and pass through, is a condition for its efficiency. This hypothesis can be found in the literature for homeless people [39]; our sample was made up of 88.3% who did not benefit from accommodation assistance, including 44.9% who slept on the street.

### Limits

Our study didn’t establish a psychiatric diagnosis. It provides information on screening and health mental care needs but we didn’t describe precisely the mental health status of asylum seekers. Finally, we were not able to compare health status of asylum seekers arriving in France with refugees living in France for longer; in order to clarify the impact of administrative status and the post-migration path on the health status of these exiled populations. This could be basis for future studies.

## Conclusion

Due to the high prevalence of health disorders among asylum seekers recently arrived in France, our study reported an important need for appropriate care for this population.

The main factors linked with a poor health status seem to be a person’s sex, sleeping in a public space and coming from West Africa.

## Data Availability

The datasets used and/or analyzed during the current study are available from the corresponding author upon reasonable request.
